# Pneumonia and Anemia in Rural Midwestern Missouri: A Retrospective Analysis

**DOI:** 10.7759/cureus.75797

**Published:** 2024-12-16

**Authors:** Jenna Watts, Morgan Stewart, Tyler Stone, Shelby Mertz, John Paulson, Nova Beyersdorfer, Kerry Johnson

**Affiliations:** 1 College of Medicine, Kansas City University, Joplin, USA; 2 Mathematics, Missouri Southern State University, Joplin, USA

**Keywords:** anemia, comorbidity mortality, pneumonia, retrospective study, rural healthcare

## Abstract

Introduction: Pneumonia and anemia are prevalent medical conditions with significant implications on patient health, resulting in numerous hospitalizations and deaths. Various studies have been done on the mortality rates of pneumonia and anemia individually. However, fewer describe the mortality of patients diagnosed with pneumonia superimposed on anemia.

Methods: This retrospective study used data from electronic medical records obtained from rural Midwestern Missouri, including 9,879 patients who were admitted with either anemia or pneumonia. The primary outcome was in-hospital mortality.

Results: The study found that patients with pneumonia had a higher mortality rate of 25.8% when diagnosed with comorbid anemia compared to the baseline group of patients with pneumonia without anemia at 14%.

Conclusion: This study demonstrates higher mortality in patients with both pneumonia and anemia than either pneumonia without anemia or anemia without pneumonia.

## Introduction

Pneumonia remains a prevalent and significant medical condition at times requiring treatment within a hospital setting. Simultaneously, anemia continues to be a common disorder affecting a substantial number of individuals. In the United States alone, approximately one million people are diagnosed and treated for pneumonia in hospitals, and the disease claims the lives of over 40,000 individuals each year [1]. Conversely, anemia stands as the most prevalent blood disorder, impacting more than three million Americans annually [2].

Extensive research has focused on investigating the relationship between chronic obstructive pulmonary disease (COPD) and anemia. Studies have found the prevalence of anemia as high as 33% in patients with COPD [3]. Additionally, COPD patients face an increased susceptibility to developing pneumonia due to various physiological manifestations. These individuals often experience chronic bronchitis, accompanied by persistent mucus production. The presence of potentially pathogenic bacteria in the respiratory tract further compounds the situation, potentially leading to a higher incidence of pneumonia within this population [4].

With the emergence of the coronavirus disease in 2019 (COVID-19), pneumonia became an even more significant burden on the healthcare system. When adjusted for other cofactors, patients with anemia had a four times higher mortality rate from COVID-19 than those without anemia [5]. Furthermore, anemia was found to be an accelerating factor for the progression of COVID-19, and increases in hemoglobin enhance patient survival rates in anemic patients [6].

Despite the extensive research on both pneumonia and anemia individually, studies have yet to be conducted to evaluate the outcomes of patients who have pneumonia or anemia in the absence of the other while also contrasting those outcomes with patients who have both pneumonia and anemia. Therefore, this study seeks to evaluate the correlation between pneumonia and anemia and, potentially, how strong their relationship is in influencing patient mortality rates.

By recognizing this association, we hope to contribute valuable insights into the interplay between pneumonia and anemia. Understanding the relationship between these conditions individually and in conjunction with each other can provide critical knowledge regarding their combined effect on patient outcomes, particularly mortality rates. This research has the potential to inform clinical practice, enhancing the management and treatment strategies for patients with both pneumonia and anemia, ultimately improving their overall prognosis and well-being.

## Materials and methods

Data collection

Clinical data was obtained and evaluated from electronic medical records at Freeman Health System (FHS), a not-for-profit hospital located in Joplin and Neosho, Missouri, with a combined 435 beds. Patient outcome data was extracted from the electronic medical record based on discharge or expiration date from January 1, 2019, to December 31, 2021, excluding prior admissions. The patient population for this study consisted of patients 18 years and older diagnosed with pneumonia or anemia based on the International Classification of Diseases, Tenth Revision (ICD-10) codes listed in Table 1 and Table 2. Excluded were patients with prior admissions within the study timeframe and patients with incomplete or missing medical records. The data was obtained retrospectively; therefore, informed consent for this study was not required. It is important to note that the population served by this hospital is primarily Caucasian and encounters the highest rate of uninsured patients in the state of Missouri [7].

**Table 1:** ICD-10 codes for pneumonia ICD-10: International Classification of Diseases, Tenth Revision; SARS: severe acute respiratory syndrome; COVID-19: coronavirus disease 2019

ICD-10 codes	Diagnosis
J1000	Influenza due to other identified influenza virus with unspecified type of pneumonia
J1001	Influenza due to other identified influenza virus with the same other identified influenza virus pneumonia
J1008	Influenza due to other identified influenza virus with other specified pneumonia
J1100	Influenza due to unidentified influenza virus with unspecified type of pneumonia
J1108	Influenza due to unidentified influenza virus with specified pneumonia
J120	Adenoviral pneumonia
J121	Respiratory syncytial virus pneumonia
J122	Parainfluenza virus pneumonia
J123	Human metapneumovirus pneumonia
J1281	Pneumonia due to SARS-associated coronavirus
J1282	Pneumonia due to COVID-19
J1289	Other viral pneumonia
J129	Viral pneumonia, unspecified
J13	Pneumonia due to *Streptococcus pneumoniae*
J14	Pneumonia due to *Haemophilus influenzae*
J150	Pneumonia due to *Klebsiella pneumoniae*
J151	Pneumonia due to *Pseudomonas*
J1520	Pneumonia due to *Staphylococcus*, unspecified
J15211	Pneumonia due to methicillin-susceptible *Staphylococcus aureus*
J15212	Pneumonia due to methicillin-resistant *Staphylococcus aureus*
J1529	Pneumonia due to other *Staphylococcus*
J153	Pneumonia due to *Streptococcus*, group B
J154	Pneumonia due to other streptococci
J155	Pneumonia due to *Escherichia coli*
J156	Pneumonia due to other Gram-negative bacteria
J157	Pneumonia due to *Mycoplasma pneumoniae*
J158	Pneumonia due to other specified bacteria
J159	Unspecified bacterial pneumonia
J168	Pneumonia due to other specified infectious organisms
J17	Pneumonia in diseases classified elsewhere
J180	Bronchopneumonia, unspecified organism
J181	Lobar pneumonia, unspecified organism
J188	Other pneumonia, unspecified organism
J189	Pneumonia, unspecified organism
J84116	Cryptogenic organizing pneumonia
J851	Abscess of lung with pneumonia
J95851	Ventilator-associated pneumonia

**Table 2:** ICD-10 codes for anemia ICD-10: International Classification of Diseases, Tenth Revision; G6PD: glucose-6-phosphate dehydrogenase

ICD-10 codes	Diagnosis
D500	Iron deficiency anemia secondary to blood loss (chronic)
D508	Other iron deficiency anemias
D509	Iron deficiency anemia, unspecified
D510	Vitamin B12 deficiency anemia due to intrinsic factor deficiency
D513	Other dietary vitamin B12 deficiency anemia
D519	Vitamin B12 deficiency anemia, unspecified
D520	Dietary folate deficiency anemia
D528	Other folate deficiency anemias
D529	Folate deficiency anemia, unspecified
D530	Protein deficiency anemia
D531	Other megaloblastic anemias, not elsewhere classified
D538	Other specified nutritional anemias
D539	Nutritional anemia, unspecified
D550	Anemia due to G6PD deficiency
D588	Other specified hereditary hemolytic anemias
D589	Hereditary hemolytic anemia, unspecified
D590	Drug-induced autoimmune hemolytic anemia
D591	Other autoimmune hemolytic anemias
D5910	Autoimmune hemolytic anemia, unspecified
D5911	Warm autoimmune hemolytic anemia
D5919	Other autoimmune hemolytic anemia
D594	Other nonautoimmune hemolytic anemias
D598	Other acquired hemolytic anemias
D599	Acquired hemolytic anemia, unspecified
D611	Drug-induced aplastic anemia
D612	Aplastic anemia due to other external agents
D6189	Other specified aplastic anemias and other bone marrow failure syndromes
D619	Aplastic anemia, unspecified
D62	Acute post-hemorrhagic anemia
D630	Anemia in neoplastic disease
D631	Anemia in chronic kidney disease
D638	Anemia in other chronic diseases classified elsewhere
D643	Other sideroblastic anemias
D6481	Anemia due to antineoplastic chemotherapy
D6489	Other specified anemias
D649	Anemia, unspecified

Statistical analysis

**Figure 1:**
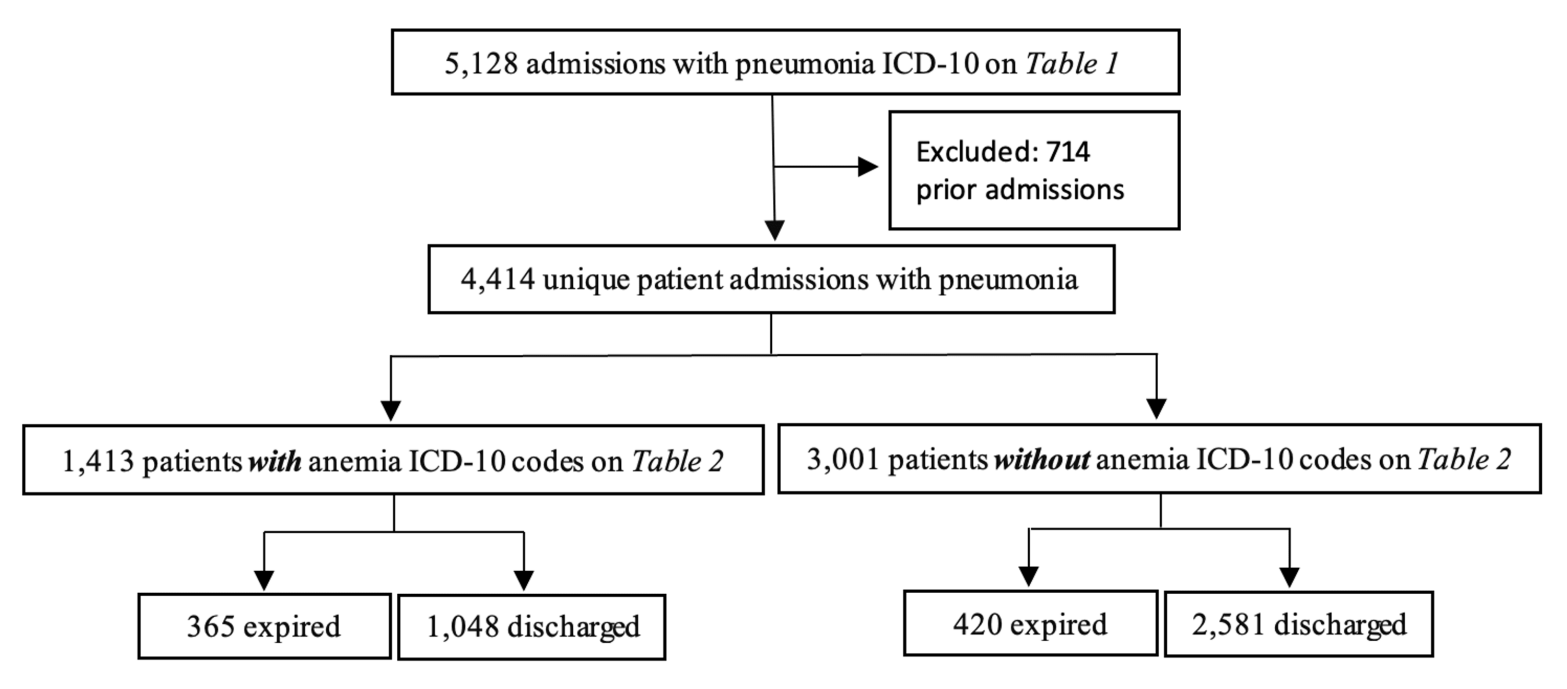
Pneumonia population separated into categories with and without anemia ICD-10: International Classification of Diseases, Tenth Revision

**Figure 2:**
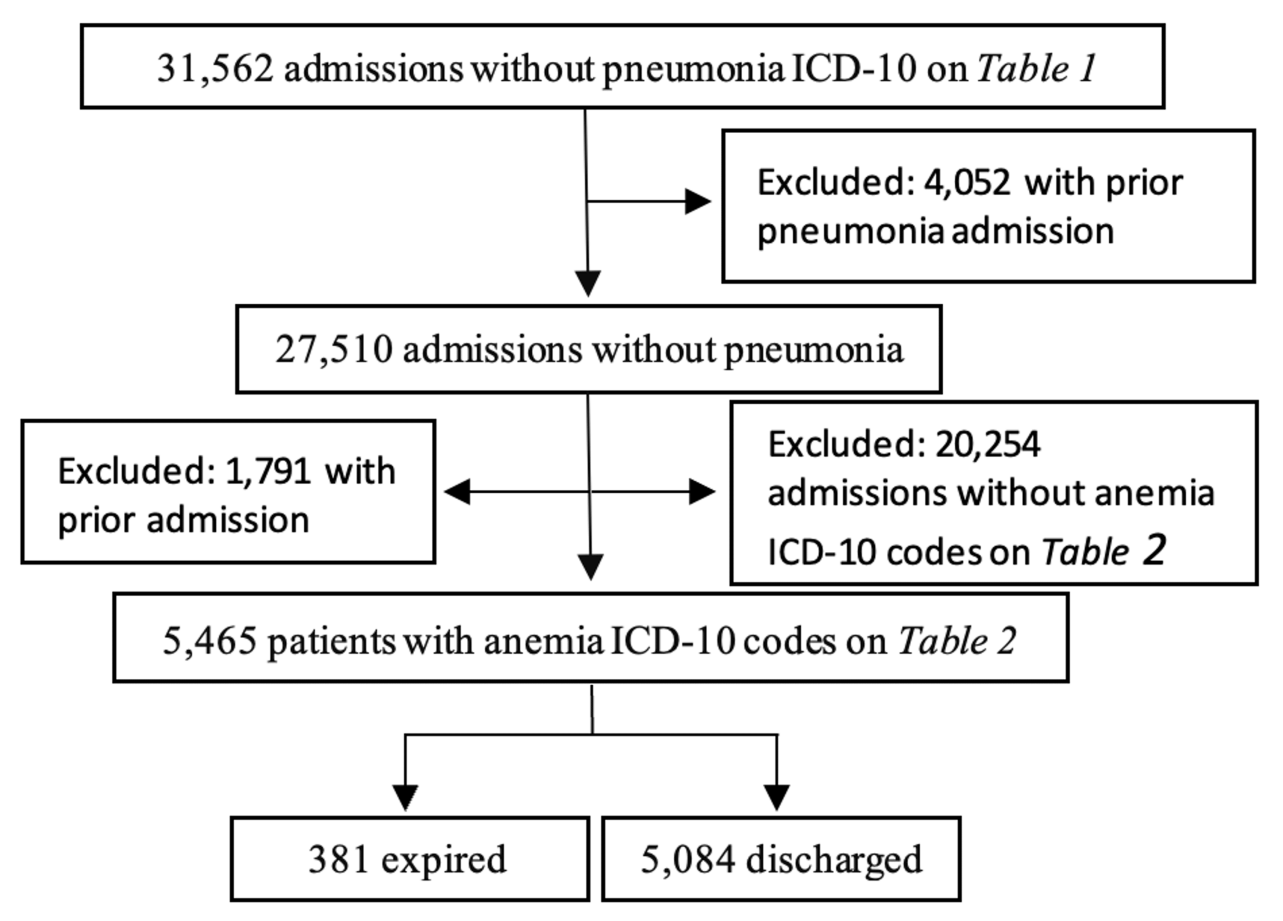
Population of those without pneumonia separated into categories with and without anemia ICD-10: International Classification of Diseases, Tenth Revision

The initial dataset consisted of 5,128 patients diagnosed with pneumonia. After excluding 714 prior admissions, 4,414 unique patients with pneumonia were included in the analysis. Of these, 1,413 patients presented with both pneumonia and anemia (P1), and 3,001 patients had pneumonia without anemia (P2) (Figure 1). The initial sample of patients identified without pneumonia was 31,562 admissions, with 4,052 excluded due to prior pneumonia admissions. Among the remaining 27,510 patients, 5,465 were identified as having anemia without pneumonia (P3) (Figure 2). Statistical analyses were performed using Wald's for calculating sample proportions and two-sample proportion summary hypothesis tests for differences in proportions. Population mortality rates were compared, and data was considered statistically significant with a p-value of <0.05 with a 95% confidence interval (Cl) used in the analysis.

Ethics statement

Approval to conduct the study was obtained from the Freeman Health System Institutional Review Board (approval number: 2022003).

## Results

Population mortality rates were compared using a two-sample proportion summary hypothesis test where statistically significant differences were found between the following groups: pneumonia with anemia group and pneumonia without anemia group (p<0.0001), pneumonia with anemia group and anemia without pneumonia group (p<0.0001), as well as pneumonia without anemia group and anemia without pneumonia group (p<0.0001) (Table 3).

**Table 3:** Two-sample proportion comparisons of mortality rates P1: patients with pneumonia and anemia; P2: patients with pneumonia without anemia; P3: patients with anemia without pneumonia; CI: confidence interval Sample 1 represents the population listed first in the comparison. Sample 2 represents the population listed second in the comparison. Sample 1 vs sample 2 shows the difference in mortality rates between the two samples being compared. The statistical test performed is a two-sample proportion test with upper and lower 95% CI calculated.

Comparison	Mortality sample 1	Mortality sample 2	Sample 1 vs sample 2	Lower 95% CI for P1-P2	Upper 95% CI for P1-P2	P-value
P1 vs P2	365 of 1413	420 of 3001	11.84%	9.24%	14.43%	<0.0001
	25.83%	14%				
P1 vs P3	365 of 1413	381 of 5465	18.86%	16.48%	21.24%	<0.0001
	25.83%	6.97%				
P2 vs P3	420 of 3001	381 of 5465	7.02%	5.61%	8.44%	<0.0001
	14%	6.97%				

**Figure 3:**
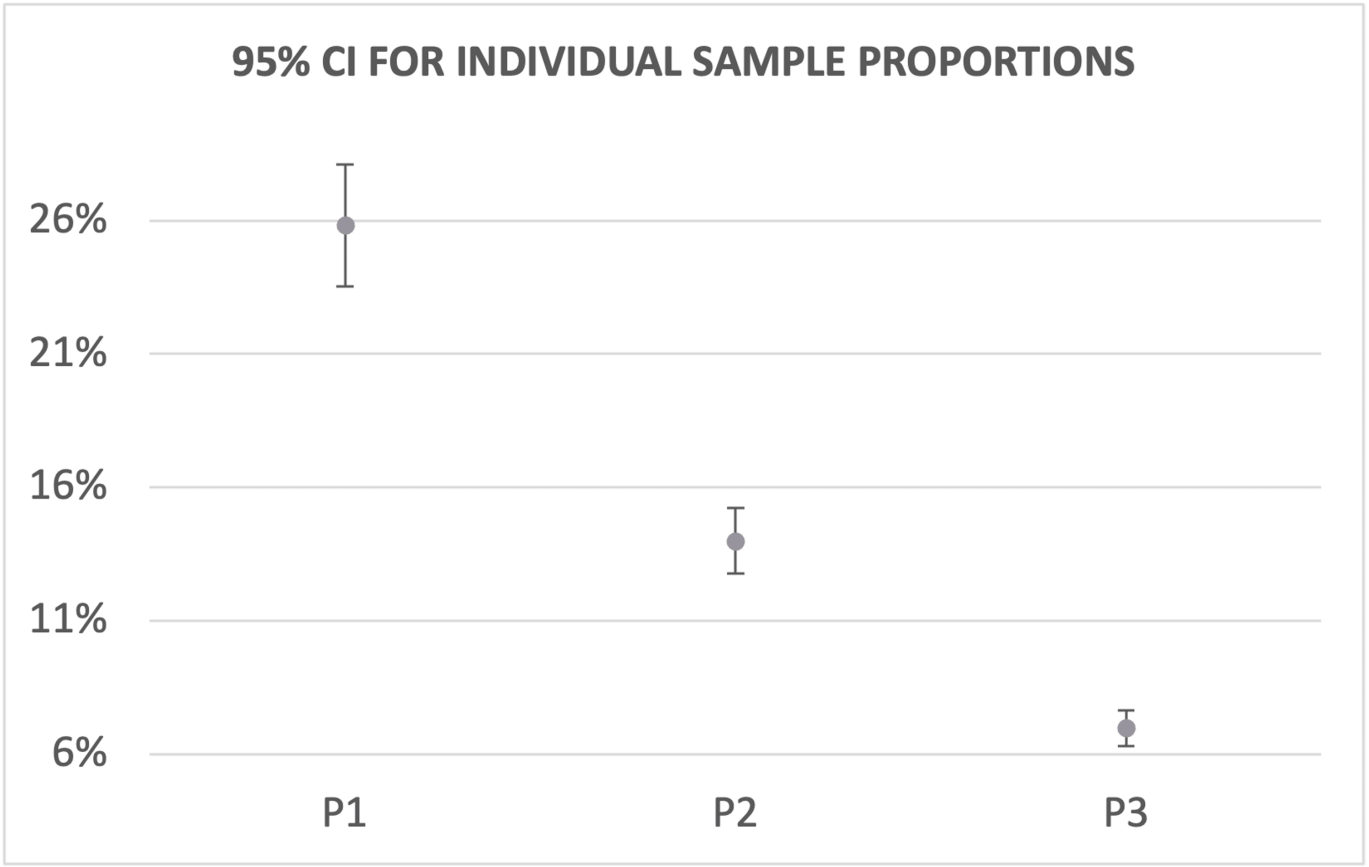
Mortality rates of population groups with CI P1: patients with pneumonia and anemia; P2: patients with pneumonia without anemia; P3: patients with anemia without pneumonia; CI: confidence interval

Patients with both pneumonia and anemia (P1) exhibited the highest mortality rate, followed by pneumonia without anemia (P2), and the lowest rate was observed in patients with anemia without pneumonia (P3) (Figure 3). Comparative analysis demonstrated that mortality rates for all three populations were significantly different. Specifically, P1 exhibited a mortality rate 9.24-14.43% higher than P2 and 16.48-21.24% higher than P3 (Table 3). P2 showed a mortality rate 5.61-8.44% higher than P3 (Table 3). These findings indicate that pneumonia is associated with higher mortality rates, particularly when combined with anemia.

## Discussion

This study was performed to determine if anemia is associated with increased pneumonia mortality rates. The finding that individuals with both conditions exhibit markedly increased mortality rates highlights the potential additive or synergistic impact of these diseases on physiological stress. Hypoxia, already a hallmark of pneumonia, may be compounded by anemia due to impaired oxygen delivery to tissues [8]. These interactions could exacerbate the severity of respiratory distress and systemic complications, leading to poorer outcomes.

In this study, all forms of anemia, including microcytic, normocytic, and macrocytic, were evaluated in our analysis. While anemia can arise from various underlying causes, such as iron deficiency, chronic disease, or vitamin B12 deficiency, this study did not differentiate between the specific types or etiologies of anemia [9]. The primary focus was on the presence of anemia as a comorbid condition and its association with increased mortality when present in patients with pneumonia. Further research may be needed to evaluate whether specific types or causes of anemia contribute differently to the outcomes observed in patients with pneumonia and anemia.

Additionally, the population served by the rural hospitals in this study was primarily Caucasian and included a high percentage of uninsured patients [10]. Socioeconomic factors such as limited access to healthcare resources and delayed medical intervention could also play a role in the outcomes observed. Future studies should seek to incorporate more diverse populations and assess socioeconomic influences to better generalize these findings.

The implications of these findings for clinical practice are significant. Early identification and management of anemia in patients with pneumonia could be prioritized as part of hospital protocols, particularly in rural healthcare settings [11]. Interventions such as iron supplementation, erythropoiesis-stimulating agents, or blood transfusions, where appropriate, could potentially mitigate some of the risks associated with anemia in these patients [12].

This study was retrospective; therefore, the sample was not chosen randomly, and it cannot be determined if the sample analyzed is representative of the population. Another limitation of this study is the inability to utilize entire patient health records; the data for this study was obtained from a single visit and did not define the patient's medical history, limiting our knowledge of the patient's baseline condition and personal health history. The lack of patient diversity is another limitation, as the population served by the rural hospitals in this study was predominantly Caucasian. We observed that individuals with both pneumonia and anemia had a higher mortality rate than pneumonia patients without anemia; however, this is not a causative factor, and the contributing factor of age cannot be ruled out at this time.

Finally, this study underscores the importance of further research to understand the interplay between anemia and pneumonia, particularly through prospective studies that can control for confounding factors like age and pre-existing comorbidities. Investigating the biological mechanisms underpinning the observed association, as well as the potential benefits of targeted treatments, could provide deeper insights into improving patient outcomes.

## Conclusions

This study demonstrates higher mortality in patients with pneumonia and anemia than pneumonia without anemia and anemia without pneumonia. These findings indicate that pneumonia is associated with higher mortality rates, particularly when combined with anemia. Understanding the interplay between these conditions provides valuable insights for clinical practice, informing management and treatment strategies to improve outcomes and overall prognosis for patients with pneumonia and anemia. In conclusion, despite the limitations of this retrospective study, it underscores the need for further research to comprehensively explore the relationship between pneumonia and anemia, including any additional factors that may contribute to the increased patient mortality. Such investigations can guide healthcare practitioners in developing tailored interventions to enhance patient care and outcomes in this vulnerable population.

## References

[1] American Thoracic Society. (2023). Top 20 pneumonia facts. Top 20 Pneumonia Facts.

[2] (2023). Anemia. https://www.hematology.org/education/patients/anemia.

[3] Sarkar M, Rajta PN, Khatana J (2015). Anemia in chronic obstructive pulmonary disease: prevalence, pathogenesis, and potential impact. Lung India.

[4] Restrepo MI, Sibila O, Anzueto A (2018). Pneumonia in patients with chronic obstructive pulmonary disease. Tuberc Respir Dis (Seoul).

[5] Veronese N, Segala FV, Carruba L (2023). Anemia as a risk factor for disease progression in patients admitted for COVID-19: data from a large, multicenter cohort study. Sci Rep.

[6] Asadzadeh R, Mozafari A, Shafiei E, Kaffashian M, Ahmadi I, Darvish M, Bastaminejad S (2022). On-admission anemia and survival rate in COVID-19 patients. Iran Biomed J.

[7] (2023). Missouri's uninsured population, before and after ACA. https://mcdc.missouri.edu/news/missouri-uninsured-post-aca/.

[8] Ouellette DR (2005). The impact of anemia in patients with respiratory failure. Chest.

[9] (2024). What is anemia?. https://www.pennmedicine.org/for-patients-and-visitors/patient-information/conditions-treated-a-to-z/anemia.

[10] Frakt AB (2019). The rural hospital problem. JAMA.

[11] Amano S, Ohta R, Sano C (2021). Recognition of anemia in elderly people in a rural community hospital. Int J Environ Res Public Health.

[12] Patel MS, McKie E, Steiner MC, Pascoe SJ, Polkey MI (2019). Anaemia and iron dysregulation: untapped therapeutic targets in chronic lung disease?. BMJ Open Respir Res.

